# Novel antibody binding determinants on the capsid surface of serotype O foot-and-mouth disease virus

**DOI:** 10.1099/vir.0.060939-0

**Published:** 2014-05

**Authors:** Amin S. Asfor, Sasmita Upadhyaya, Nick J. Knowles, Donald P. King, David J. Paton, Mana Mahapatra

**Affiliations:** The Pirbright Institute, Pirbright Laboratory, Ash Road, Woking, Surrey GU24 0NF, UK

## Abstract

Five neutralizing antigenic sites have been described for serotype O foot-and-mouth disease viruses (FMDV) based on monoclonal antibody (mAb) escape mutant studies. However, a mutant virus selected to escape neutralization of mAb binding at all five sites was previously shown to confer complete cross-protection with the parental virus in guinea pig challenge studies, suggesting that amino acid residues outside the mAb binding sites contribute to antibody-mediated *in vivo* neutralization of FMDV. Comparison of the ability of bovine antisera to neutralize a panel of serotype O FMDV identified three novel putative sites at VP2-74, VP2-191 and VP3-85, where amino acid substitutions correlated with changes in sero-reactivity. The impact of these positions was tested using site-directed mutagenesis to effect substitutions at critical amino acid residues within an infectious copy of FMDV O1 Kaufbeuren (O1K). Recovered viruses containing additional mutations at VP2-74 and VP2-191 exhibited greater resistance to neutralization with both O1K guinea pig and O BFS bovine antisera than a virus that was engineered to include only mutations at the five known antigenic sites. The changes at VP2-74 and VP3-85 are adjacent to critical amino acids that define antigenic sites 2 and 4, respectively. However VP2-191 (17 Å away from VP2-72), located at the threefold axis and more distant from previously identified antigenic sites, exhibited the most profound effect. These findings extend our knowledge of the surface features of the FMDV capsid known to elicit neutralizing antibodies, and will improve our strategies for vaccine strain selection and rational vaccine design.

## Introduction

Foot-and-mouth disease (FMD) remains one of the most economically important diseases of cloven-hoofed animals worldwide. The causative agent, FMD virus (FMDV), is an aphthovirus that belongs to the family *Picornaviridae*. It exists as seven immunologically distinct serotypes (O, A, C, Asia 1, SAT 1, SAT 2 and SAT 3). The disease is widespread across the world, especially in Africa and Asia, and is able to spread rapidly (Alexandersen *et al.*, 2003). Serotype O is the most common cause of outbreaks globally followed by serotype A (Ayelet *et al.*, 2009; Borley *et al.*, 2013; Rweyemamu *et al.*, 2008). In addition to circulation of FMD in endemic regions, there are occasional incursions into countries that are normally disease-free, e.g. in the UK in 2001 and in Japan in 2011, resulting in very high economic losses.

The virus consists of a non-enveloped capsid of icosahedral symmetry containing a single-stranded positive-sense RNA genome. A single ORF encodes all the capsid proteins (VP1–4), together with a total of nine additional mature, non-structural proteins, including two proteases (L and 3C) and an RNA-dependent RNA polymerase (3D). Only VP1, VP2 and VP3 are exposed on the outer surface of the virus particle and, thus, these three proteins determine both the specificity of virus-neutralizing antibodies and also the ability of the virus to interact with its receptors on host cells. The three-dimensional structures of several serotypes of FMDV have been determined (Acharya *et al.*, 1989; Lea *et al.*, 1994; Logan *et al.*, 1993) and immunological epitopes have been mostly found to be located on surface-oriented interconnecting loops between structural elements. Significant antigenic diversity within serotypes limits the ability of vaccines to provide cross-protection against diverse field strains (Brehm *et al.*, 2008; Maree *et al.*, 2011; Paton *et al.*, 2005; Reeve *et al.*, 2010). A vaccine providing broader antigenic coverage would be a valuable tool for the control of FMD.

**Fig. 1.**
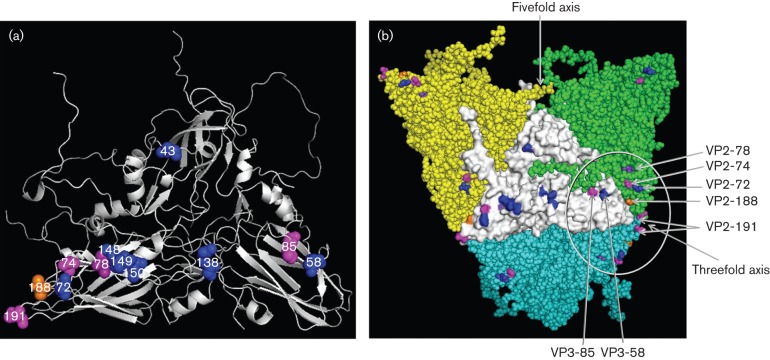
(a) O1K reduced structure (cartoon) showing position of critical amino acid residues of five neutralizing antigenic sites in blue. Residue VP2-188 shown in orange and VP2-78 shown in purple have been identified using bovine mAbs (Barnett *et al.*, 1998). Additional residues mutated in this study are shown in magenta. (b) Three-dimensional structure (external surface) of the O1K (reduced) showing changes in antigenic sites 2 and 4 within the circle. Changes in amino acids forming the five neutralizing antigenic sites (5M) in the central protomer (shown in white) are shown in blue. Amino acids at position VP3-85 (close to VP3-58 that defines antigenic site 4), VP2-191 (located at the threefold axis) and VP2-74 (close to VP2-72 that defines antigenic site 2) are shown in magenta, VP2-188 in orange and VP2-78 in purple.

The role of antibody as the principal component of the immune response to FMDV infection is well established (Juleff *et al.*, 2009; Pay & Hingley, 1987). Several studies have been carried out in the past two decades to identify the targets on the virus surface for the binding of neutralizing antibodies, and monoclonal antibodies (mAbs) have been powerful tools for identifying the amino acid footprint of different antigenic sites, mainly by sequencing mAb-resistant (mar) mutants. This approach has been used successfully to delineate the neutralizing antigenic sites of viruses representing different serotypes (Aktas & Samuel, 2000; Baxt *et al.*, 1989; Crowther *et al.*, 1993; Kitson *et al.*, 1990; Mahapatra *et al.*, 2011; Maree *et al.*, 2013; Mateu *et al.*, 1990; Xie *et al.*, 1987). FMDV serotype O has been studied most extensively, where five neutralizing antigenic sites (1–5) have been identified on its surface, involving three of the capsid proteins (VP1–3). Site 1 is linear and trypsin sensitive, whereas all the other identified sites are conformational and trypsin resistant. For serotype O viruses, the G–H loop and carboxy-terminus of VP1 contribute to site 1, with critical residues that affect antibody binding at positions 144, 148 and 150, and 208. Critical amino acids at positions 70–73, 75, 77 and 131 of VP2 contribute to site 2, and for site 3, these residues are at 43 and 44 of the B–C loop of VP1. Only one critical residue, at position 58 of VP3, has been identified for site 4. The fifth site, characterized by an amino acid at position 149 of VP1, is probably formed by interaction of the VP1 G–H loop region with other surface amino acids. (Aktas & Samuel, 2000; Barnett *et al.*, 1998; Crowther *et al.*, 1993; Kitson *et al.*, 1990; McCahon *et al.*, 1989; Xie *et al.*, 1987) (Fig. 1).

A mutant virus resistant to neutralizing murine mAbs to sites 1–5 (5-site mutant virus) was reported to resist neutralization by bovine polyclonal sera raised against the parent virus (Dunn *et al.*, 1998). Nonetheless, guinea pigs were protected from challenge by virulent wild-type (wt) parent and mutant viruses after immunization with either wt or mutant 146S antigen as inactivated whole-virus vaccines/hyperimmune sera. Thus the antigenic sites could be larger than the epitopes recognized by individual mAbs. This also indicates the existence of other undisclosed protective epitopes in FMDV, the identity of which has been explored in the present study, using a reverse genetics approach. This study provides evidence for the existence of a new neutralizing epitope that involves VP2-191 at the threefold axis of the capsid, which extends our knowledge of the surface features of the FMDV capsid known to elicit neutralizing antibodies, and also could help better predict vaccine matching and the development of more broadly cross-reactive vaccines.

## Results

### Generation of full-length genome plasmids with mutations

Plasmid pT7S3 containing the full-length FMDV O1K cDNA was modified to introduce unique restriction sites for easy switching of the capsid coding region (Fig. S1 available in the online Supplementary Material). The deletion of the *Spe*I site in the vector sequence and the introduction of the *Spe*I in the 2B region using inverse PCR primers did not change the encoded amino acid sequences. In this study, we have produced a quintuple mutant virus (5M, Table 1a) by cloning the capsid of the quadruple mutant, G67 virus and adding another mutation to substitute the amino acid at VP1-149, making it identical to the quintuple mutant previously created by Crowther *et al.* (1993) (Table 1b). The pT7S3/5M plasmid was used as the template to introduce further mutations in the capsid coding regions in addition to the five known neutralizing antigenic sites. Three residues (VP2-74, 191 and VP3-85) were selected for this purpose as they were indicated to have an impact on the antigenicity of the virus by comparison of capsid sequences with *in vitro* virus cross-neutralization data and also by epitope prediction using viral crystal structure (Borley, 2013; Borley *et al.*, 2013). In addition, these residues, especially VP2-74 and VP2-191, are variable among serotype O viruses, whereas the adjacent residues are highly conserved (Fig. S2) indicating serotype O FMDV can tolerate mutation at these positions. Changes at these three positions were engineered in the 5M infectious copy plasmid, resulting in the generation of five additional single mutants and three additional double mutants (Table 1a, Fig. 1a, b). A single mutant (O1K-VP2-191) was also made by introducing a mutation at codon position VP2-191 in the pT7S3/O1K-wt plasmid. In addition, two full-length genome plasmids containing 26 mutations in the capsid residues (O1K-wt26n and O1K-wt26a) were generated (Table 1c). The capsid coding regions of all the plasmids were sequenced on both the strands and no unwanted mutations were observed.

**Table 1(a).**
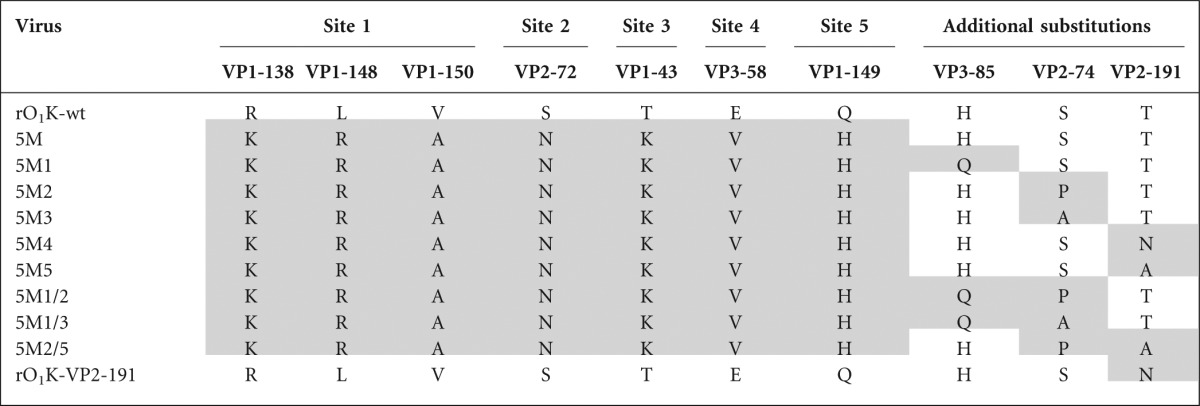
List of O1 Kaufbeuren mutant viruses generated in this study and their associated amino acid substitutions Positions different from the rO1K-wt virus are shaded.

**Table 1(b).** Residue changes in mar-mutant viruses that resisted neutralization by specific mAb combinations

Virus	mAbs used	Capsid amino acid substitutions
G67	D9, C8, C6, 14EH9 (sites 1–4)	VP1-43, 138, 148, 150, VP2-72, VP3-58
G67+OC3	D9, C8, C6, 14EH9, OC3 (sites 1–5)	VP1-43, 138, 148, 149, 150, VP2-72, VP3-58

**Table 1(c).** Capsid amino acid residue positions which were substituted in commercially synthesized clones

Virus	VP1												
Amino acid position												
	43	45	96	138	139	142	148	149	150	197	198	208	210
rO1K-wt	T	Q	K	R	N	P	L	Q	V	T	E	P	K
rO1K-wt25n	K	K	T	D	R	A	R	H	A	N	Q	L	E
rO1K-wt25a	A	A	A	A	A	A	A	A	A	A	A	A	A

	VP2										VP3		
Amino acid position											Amino acid position		
	70	71	72	73	74	75	77	79	131	134	58	60	174
rO1K-wt	V	T	S	D	S	F	R	H	S	K	E	G	V
rO1K-wt25n	F	N	N	N	Q	L	H	Y	P	E	V	H	T
rO1K-wt25a	A	A	A	A	A	A	A	A	A	A	A	A	A

### Rescue and characterization of FMDV from full-length genome plasmids

Following electroporation, live infectious viruses were successfully recovered from all the cDNA clones except the two (pT7S3/O1K-wt26n and pT7S3/O1K-wt26a) containing 26 amino acid substitutions in the capsid. FMDV-specific cytopathic effects (CPE) were observed 24–36 h post-electroporation. Extensive CPE were observed at both the first and second passages. At least two independent clones of each virus were rescued. However, only one clone in each case was used for further characterization. In order to prove that the expected viruses had been rescued, reverse transcriptase (RT)-PCR was carried out on the RNA extracted from infected BHK-21 cells using primer pair O1K-AflIIF and O1K-SpeIR that produced a 2500 bp-long fragment (encompassing the C-terminal part of L, P1, 2A and the N-terminal part of 2B; Fig. S2) of expected size (data not shown). No PCR products were generated in parallel reactions in which the enzyme reverse transcriptase was omitted, indicating that the amplified products were not generated from the transfected plasmid DNA. The PCR products were sequenced on both the strands and they contained no additional mutations. The genetic conformations of these viruses were stable on passage in cell culture and no reversion was noticed after sequential passages of the recombinant viruses for at least four passages in BHK-21 cells.

### Growth kinetics and phenotypic characterization of the recombinant viruses

Standard multi-step growth curves were carried out to compare the growth of the recombinant viruses with the parent viruses. All the viruses grew at a similar rate and to a similar titre, indicating the mutations in the antigenic sites had no adverse effects on the replication efficiency of these viruses *in vitro* (Fig. S3). BHK-21 cells infected with the parent or recombinant viruses were stained following infection, and photographed. Both the parent and the recombinant viruses exhibited variable size plaques with no clear differences between them (data not shown).

### Binding of site-specific mAbs and guinea pig antisera with the recombinant viruses

**Fig. 2.**
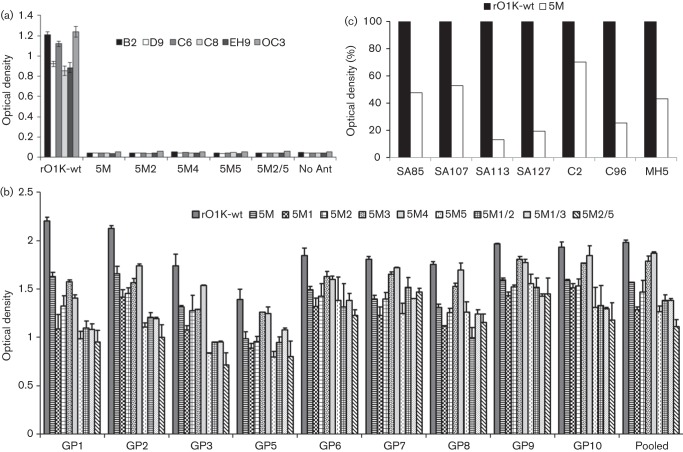
(a) Reactivity profile (ELISA) of rO1K-wt virus and its derivatives with neutralizing antigenic site-specific O Lausanne murine mAbs. Sucrose gradient-purified 146S antigens were used to ensure all the mutant viruses had an equivalent amount of antigen in the test. (b) ELISA results of individual and pooled guinea pig hyperimmune sera against rO1K-wt virus and its derivatives. Sucrose gradient-purified 146S antigens were used to ensure all the mutant viruses had an equivalent amount of antigen in the test. (c) Reactivity profile (ELISA) of the 5M virus when compared to the parent rO1K-wt virus using neutralizing antigenic site-specific O Manisa murine mAbs (SA85, SA107, SA113 and SA127) and O Lausanne bovine mAbs (C2, C96 and MH5). Sucrose gradient-purified antigens were used to ensure all the mutant viruses had an equivalent amount of antigen in the test.

An indirect ELISA was carried out to study the binding of the antigenic site-specific mAbs with the parent rO1K-wt and the mutant viruses. As expected, all the mAbs used to generate the mar-mutant virus (B2, D9, C6, C8, EH9 and OC3) recognized the epitopes on the parent virus whereas none of the mAbs bound to the mutant viruses (Fig. 2a), confirming the abrogation of mAb binding in the mutant viruses. In the ELISA, pooled and individual guinea pig antisera were able to react to varying degrees with the recombinant viruses as well as all the rO1K-wt virus (Fig. 2b), suggesting the existence of other unidentified neutralizing and/or non-neutralizing epitopes.

### Binding of neutralizing antigenic site-specific murine and bovine mAbs with 5M virus

The 5M virus did not react with any of the O Lausanne murine neutralizing antigenic site-specific mAbs that were used to generate the mar-mutant viruses. Further, we studied the reactivity of this virus with other available well-characterized neutralizing murine and bovine mAbs (in ELISA) to determine whether there were neutralizing epitopes accessible for binding of mAbs recognizing residues in the same region. Unfortunately, there were no additional mAbs available against antigenic site 1. Some of the mAbs recognizing antigenic site 2 exhibited substantial residual binding with the 5M virus (Fig. 2c), indicating availability of epitopes for antibody binding. A similar result was observed for antigenic site 3-specific bovine mAb, MH5. Therefore mutation of further amino acid residues in these antigenic sites may enhance resistance to neutralization.

### Virus-neutralizing antibody (VN) titres

**Fig. 3.**
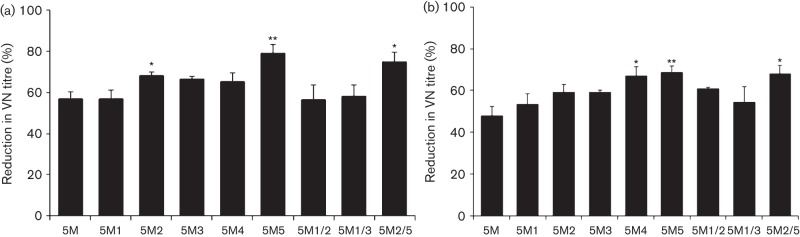
(a) Percentage reduction in VN titre of the single and double mutant viruses when compared to the parent rO1K-wt virus using guinea pig antisera. Two-dimensional VN testing was carried out using post-vaccinal sera raised in guinea pigs against rO1K-wt vaccine antigen. * and ** indicate significant difference (to 5M virus) at *P*<0.05 and *P*<0.01, respectively. (b) Percentage reduction in VN titre of the single and double mutant viruses when compared to the parent rO1K-wt virus using bovine antisera. Two-dimensional VN testing was carried out using post-vaccinal sera raised in bovine against a similar type O antigen (O BFS). * and ** indicate significant difference (to 5M virus) at *P*<0.05 and *P*<0.01, respectively.

The main aim of this study was to quantify the reduction in neutralization following mutations in the antigenic sites of FMDV. Therefore, it was crucial to determine the VN titre of the sera against all the mutant viruses at a fixed virus dose (100 TCID_50_), for which a two-dimensional micro-neutralization test was carried out using five different doses of the virus encompassing 100 TCID_50_ for this purpose. The resultant VN titres at each virus dose were used to calculate the serum titre at 100 TCID_50_ by regression analysis, and the results using pooled guinea pig antisera are shown in Fig. 3(a). Compared with the parent rOIK-wt, there was a 57 % reduction in VN titre against the 5M virus where the critical residues of all five known neutralizing antigenic sites had been substituted. Surprisingly, the 5M1 virus exhibited a similar reduction in VN titre as the 5M virus, indicating that mutation at VP3-85, which is close to antigenic site 4, did not have any additional impact on the efficiency of neutralization. However, the addition of other single substitutions either at VP2-74 or VP2-191 resulted in a further 10–25 % reduction in VN titre, substitution at VP-191 having the greater impact. Substitution of serine at VP2-74 by proline (5M2) or alanine (5M3) essentially had a similar effect on VN titre. Interestingly, substitution at VP2-191 by alanine (5M5) was 15 % more efficient at reducing neutralization than substitution by asparagine (5M4).

Double mutations were introduced into the 5M virus to find out whether neutralization could be further reduced. However, introduction of mutations at VP3-85 and VP2-74 (5M1/2 and 5M1/3) and at VP2-74 and VP2-191 (5M2/5) had no additional effect on the efficiency of neutralization of the resultant mutant viruses (Fig. 3a).

As bovine sera against the parental rO1K-wt virus were not available, sera against a very closely related vaccine strain, O BFS, were used to study the impact of the mutations on neutralization by antibodies from a FMD target species. O BFS is 99.2 % identical to O1K-wt at the capsid amino acid level and has the identical amino acid residues as O1K-wt at the critical positions described for the five neutralizing antigenic sites. Analysis of the VN data revealed similar findings as with guinea pig sera, with a 48 % drop in neutralization for the 5M virus, a further 10–20 % diminution with additional single mutations, and no additional impact resulting from double mutations (Fig. 3b).

### Characterization of rO1K-VP2-191 with guinea pig polyclonal sera and neutralizing antigenic site 2-specific mAbs

**Fig. 4.**
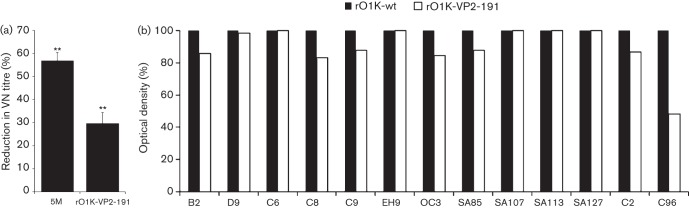
(a) Percentage reduction in VN titre of the rO1K-VP2-191virus when compared to the parent rO1K-wt virus. Two-dimensional VN testing was carried out using the guinea pig antisera raised against rO1K-wt viral antigen. ** indicates significant difference at *P*<0.01. (b) Reactivity profile (ELISA) of rO1K-VP2-191 virus with neutralizing antigenic site-specific O Lausanne murine mAbs (B2, D9, C6, C8, EH9 and OC3), neutralizing antigenic site 2-specific O Manisa murine mAbs (SA85, SA107, SA113 and SA127) and O Lausanne bovine mAbs (C2 and C96). Sucrose gradient-purified 146S antigens were used to ensure all the mutant viruses had an equivalent amount of antigen in the test.

The rO1K-VP2-191 virus was generated to determine the involvement of VP2-191 with neutralizing antigenic site 2. This virus exhibited a 30 % reduction in VN titre when compared with the parent rO1K-wt virus using guinea pig polyclonal sera (Fig. 4a). It also reacted well with all seven O Lausanne murine mAbs (Table 2a) representing neutralizing antigenic sites 1–5 used in this study (Fig. 4b), indicating the substitution at position VP2-191 did not have any significant effect on the binding of mAbs to those particular epitopes, including C6 and C9, that are directed towards antigenic site 2 and are closest to VP2-191 (Fig. 1b). The reactivity of this virus with other neutralizing antigenic site 2-specific O Manisa murine mAbs (Table 2a) was studied using ELISA and was found to be similar as the parent rO1K-wt virus (Fig. 4b).

**Table 2(a).** List of mAbs used in this study and their respective critical residue positions

mAb	Critical residue
*O Lausanne murine*	
B2 (site 1)	VP1 144, 148, 150
D9 (site 1)	VP1 148
C6 (site 2)	VP2 73
C8 (site 3)	VP1 43, 44
C9 (site 2)	VP2 73
14 EH9 (site 4)	VP3 58
OC3 (site 5)	VP1 149
*O Manisa murine*	
SA85 (sites 2 and 5)	VP2 72, VP1 149
SA107 (site 2)	VP2 73
SA113 (site 2)	VP2 72
SA127 (site 2)	VP2 73
*O Lausanne bovine*	
C2 (site 2)	VP2 188
C96 (sites 2 and 4)	VP2 78, VP3 58
MH5 (site 3)	VP1 46

### Reactivity of recombinant viruses with bovine mAbs

**Fig. 5.**
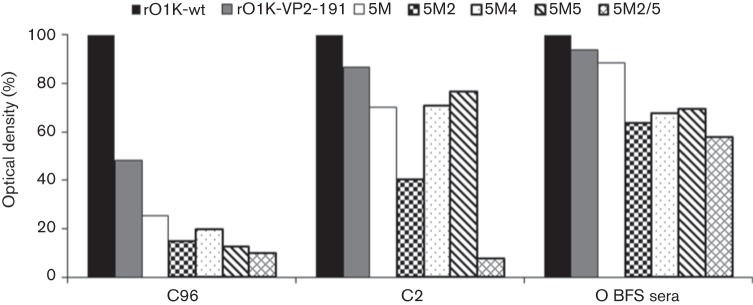
Reactivity profile (ELISA) of the single and double mutant viruses when compared to the parent rO1K-wt virus using site 2-specific O Lausanne bovine mAbs. Sucrose gradient-purified 146S antigens were used to ensure all the mutant viruses had an equivalent amount of antigen in the test; an O BFS post-vaccinal serum was used as a positive control.

The reactivity profile of the mutant viruses with two bovine mAbs, C96 and C2, was studied and the results are presented in Fig. 5. These mAbs have been reported to abrogate binding of mouse mAbs directed towards neutralizing antigenic sites 2 and 4 (Barnett *et al.*, 1998). The rO1K-VP2-191 virus exhibited an intermediate level of reactivity with mAb C96 (critical residues: VP2-78 and VP3-58), whereas the rest of the recombinant viruses reacted poorly with this mAb (Fig. 5). Surprisingly, all the recombinant viruses reacted well with mAb C2 (critical residue: VP2-188), except the 5M2 and 5M2/5 viruses, which showed intermediate or poor reactivity, respectively. This indicates the importance of the mutation at VP2-74 to binding of mAb C2 and also that the effect of a mutation at VP2-191 only becomes apparent after binding at VP2-74 has been impaired.

### Threshold of neutralization in serotype O FMDV

**Fig. 6.**
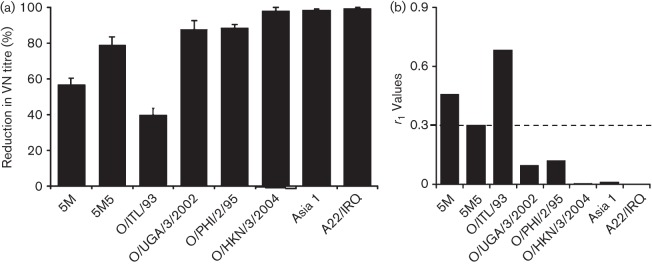
Percentage reduction in VN titre (a) and the associated *r*_1_ values (b) of various serotypes O, A and Asia 1 field isolates in comparison to the rO1K-wt virus. Two-dimensional VN testing was carried out using the guinea pig antisera raised against rO1K-wt viral antigen.

To determine the threshold of reduction in VN titre of the guinea pig sera, VN tests were also carried out using diverse field isolates from serotype O belonging to topotypes other than Europe–South America represented by O1K (Table 2b) and other serotypes (A and Asia 1). The VN titre reductions and their corresponding *r*_1_-values are presented in Fig. 6(a, b). There was 9–13 % variation at capsid amino acid level between O1K-wt and other type O viruses belonging to different topotypes, whereas 33–37 % variation was observed between serotypes (Table 2b). The O/ITL/1/93 (ME-SA topotype) originating from the same geographical area exhibited a relatively modest reduction in VN titre (39 %), whereas viruses from a distant geographical area (O/PHI/2/95-Cathay topotype or O/UGA/3/2002-East Africa 2 topotype) cross-reacted less, giving rise to an 88 % reduction in VN titre. Interestingly, for the Cathay topotype virus, O/HKN/3/2004 of porcine origin, there was a 98 % reduction in VN titre, although both the viruses from the Cathay topotype had about 13 % variation in capsid amino acid sequence. Similarly, both O/ITL/1/93 and O/UGA/3/2002 had 9.8 % variation in capsid amino acid sequence, but exhibited 39 % and 87 % reduction in VN titre, respectively, indicating that the position of the amino acid changes are more important in terms of antigenicity of the virus than mere number. As expected, viruses from another serotype were not neutralized at all. A similar analysis using the O BFS bovine sera was not possible because of the limited amount of material available for this study.

**Table 2(b).**
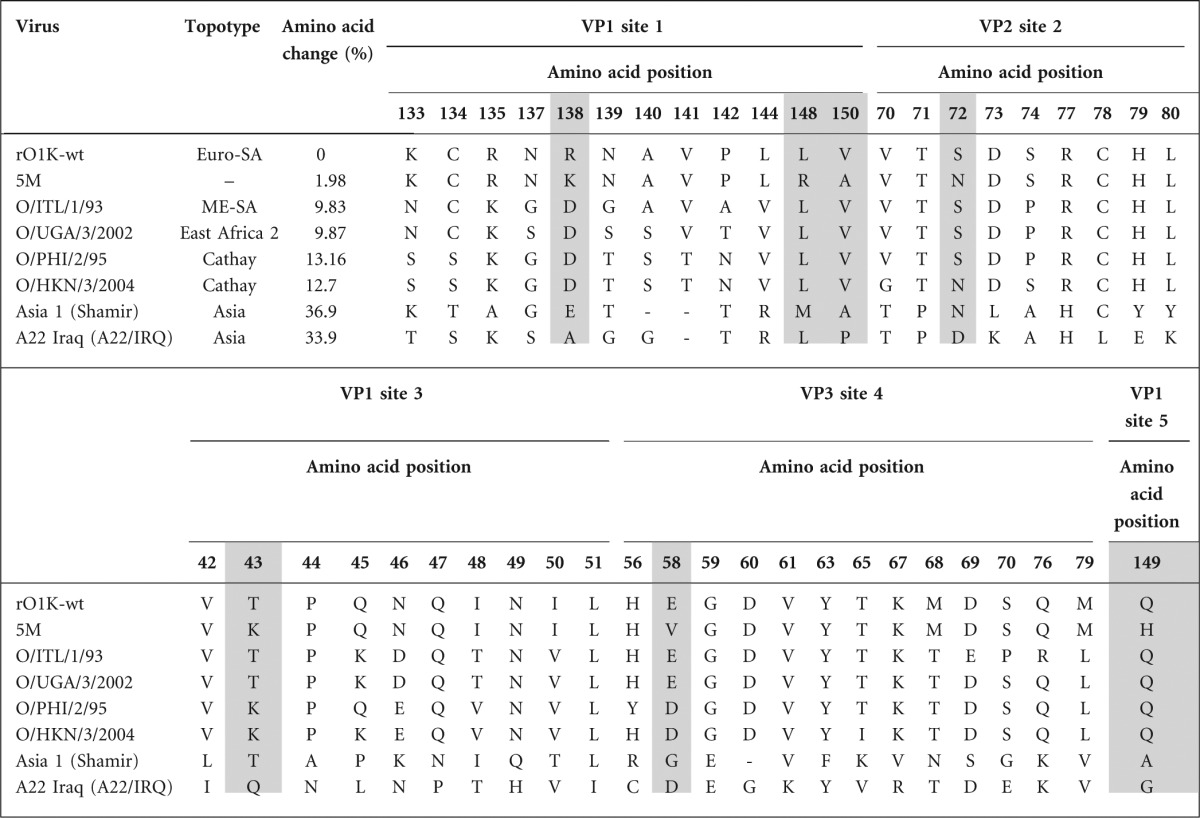
Amino acid changes between rO1K-wt virus and other viruses from different topotypes and serotypes in the five neutralizing antigenic site regions The residue positions where the 5M virus has a substitution are shaded. –, Deletion of amino acid at equivalent positions. The GenBank accession numbers for the FMDV capsid sequences are: O1K, X00871; Asia 1 Shamir, JF739177; A22, AY593763; O/ITL/1/93, KJ415244; O/UGA/3/2002, KJ415246; O/PHI/2/95, KJ415245 and O/HKN/3/2004, KJ415243.

## Discussion

The mechanism of protection afforded by viral vaccines is not very well understood, although the critical role of neutralizing antibodies in protection induced by FMD vaccination is well established. In the case of FMDV (O1K), a quintuple mutant (G67+OC3) was generated by successive single-step selection with mAbs involving neutralizing antigenic sites 1–5 (Crowther *et al.*, 1993; McCahon *et al.*, 1989). Capsid sequence analysis of this mutant revealed a total of seven amino acid substitutions in the capsid, and the virus was reported to be resistant to polyclonal serum from cattle vaccinated with O1K virus. Dunn *et al.* (1998) used this quintuple mutant to explore the contribution of these neutralizing epitopes to the protection provided by inactivated vaccines by cross-challenge experiments in a guinea pig model. They reported that the five-site mutant resisted *in vitro* neutralization by guinea pig antiserum raised against the parent virus (and vice versa); nevertheless, guinea pigs were protected by antibodies in two-way cross-challenge studies using the parent and mutant viruses as vaccine antigens or challenge viruses. Adoptive transfer of antiserum was used in these studies to confirm the role of antibody in the demonstrated protection. This indicated the existence of other, as-yet-unidentified, protective epitopes. In fact, analysis of their figures reveals that there was a considerable amount of residual neutralization/antibody binding detected by VN titre and ELISA, respectively, when the guinea pig antisera raised against either wt or mutant antigens were tested *in vitro*. Therefore, a recombinant copy of the quintuple mutant studied by Dunn *et al.* (1998) was first constructed and investigated for its reactivity with neutralizing murine and bovine mAbs. As expected, none of the mAbs used to select the quintuple mutant virus recognized the recombinant 5M virus. However, several other site-2 mAbs and one site-3 mAb still bound to a significant extent, indicating that these sites had not been fully abrogated by the introduced mutations. A contributory factor might be that only a single mutation had been introduced at each of the sites 2–5 in the 5M virus, whereas four mutations had been introduced in the G–H loop (site 1). Considering this, and that antigenic site 2 has been reported to be immunodominant in the polyclonal response of FMD-vaccinated animals (Mahapatra *et al.*, 2012), it is not surprising that guinea pigs were protected in cross-challenge experiments carried out by Dunn *et al.* (1998).

To completely block binding of antibodies, it was decided to mutate a total of 26 capsid amino acids (13 in VP1, 10 in VP2 and three in VP3) associated with the known antigenic sites. The capsid coding regions of the pT7S3-*Spe*I plasmid were successfully replaced with the changed residues incorporated in the capsid of two commercially synthesized DNA inserts. However, infectious virus could not be recovered from either of the constructs upon repeated electroporation, even after multiple blind passages, although live virus was recovered from the positive control plasmid each time. We concluded that the changes in the capsid coding region could not be tolerated, and instead adopted a different strategy by adding additional mutations one by one to the 5M virus to ascertain the tolerance of the virus to accommodate changes, and then studying the impact on antigenicity. To this effect, a total of five mutations were added at three different positions, (VP3-85, VP2-74 and VP2-191) either individually or in combinations to make a total of ten single or double mutants.

Some of the additional mutations added to the 5M construct increased resistance to neutralization by guinea pig or bovine antisera, but complete resistance to neutralization was not observed. The mutation introduced at VP3-85 had little effect on the efficiency of neutralization. However, VP2-74 and VP2-191 mutations increased resistance to neutralization by 15–22 % compared with the 5M virus. This change is similar to the findings of Crowther *et al.* (1993), who reported a 15 % drop in neutralizing antibody titre with post-vaccination cattle sera following a single mutation to make the quintuple mutant from its quadruple mutant precursor (G67 to G67+OC3 mutant). The substitution of VP2-74 from serine to proline/alanine had a similar impact on neutralization in both guinea pig and bovine antisera, whereas the substitutions at position VP2-191 had a more prominent effect with guinea pig antisera. Substitution at this position from a threonine to an alanine had the most pronounced effect on resistance to neutralization, which may be explained by this change being from a hydrophilic to a hydrophobic residue whereas the other change was between two hydrophilic residues. Unexpectedly, the double mutant (5M2/5) containing both VP2-74 and VP2-191 mutations did not show a greater resistance to neutralization than the individual counterparts.

VP2-191 is located at the threefold axis of the capsid, close to VP2-188 (only 9.6 Å apart). VP2-188 has an intermediate position between VP2-191 and antigenic site 2 (VP2-70-79), VP2-72 being 5.6 Å away from VP2-188 versus 17 Å from VP2-191. VP2-188 has been reported to be a part of antigenic site 2, based upon studies using bovine mAbs (Barnett *et al.*, 1998). Therefore, the question arises whether VP2-191 is also part of antigenic site 2 or constitutes another epitope independent of antigenic site 2. To resolve this, another variant was generated from the parent rO1K-wt virus by introducing a T to N substitution at position VP2-191. An aspargine substitution was selected, as in serotype O field isolates this residue occurs in order of frequency T>N>S. A significant reduction in neutralization was observed, confirming the involvement of VP2-191 in the neutralization process. The reactivity of rO1K-VP2-191 virus was profiled with antigenic site 2-specific mAbs to investigate the link between VP2-191 and antigenic site 2. This virus reacted with all the site 2-specific murine mAbs to the same extent as the parent rO1K-wt virus, indicating this amino acid is not involved in the epitopes recognized by these murine mAbs. In contrast, the poor reactivity of the bovine mAb C96 with the 5M virus and all its derivatives implied that the changes induced by the substitutions at positions VP2-72 and VP3-58 were enough to abrogate its binding. Additional mutations at position VP2-74 or VP2-191 did not result in further reduction in binding of the mAb. Surprisingly, substitutions at VP2-72 or VP2-191 did not have much impact on the binding of the C2 mAb, indicating the changes induced by these mutations were not enough to abrogate binding of the antibody. However, a substitution at VP2-74 (S to P) resulted in a significant reduction in antibody binding. This may be explained by the more profound epitope disturbance caused by a substitution with proline that is well known as a helix breaker. The recombinant 5M2/5 virus containing an additional substitution at VP2-191, as well as VP2-74, resulted in complete abrogation of the antibody binding to this epitope. Though initial experiments with the murine mAbs indicated that VP2-191 could be an independent epitope/site, experiments with the bovine mAbs confirmed that it is in some way linked to neutralizing antigenic site 2.

Introduction of the additional mutations to the 5M virus resulted in a final reduction in neutralization titre of 80 % or 70 % relative to the wild-type virus, using guinea pig or bovine sera, respectively, which is about 20 % more than seen with the 5M mutant virus. The ability of the same guinea pig antiserum to neutralize a range of diverse FMDV isolates was investigated to study threshold of neutralization inter- and intra-serotypically. With O/UGA/3/2002 virus (EA-2 topotype) there was an 88 % reduction in neutralizing antibody titre, whereas the titre reduction with O/ITL/1/93 (ME-SA topotype) was less than half (~40 %) although both of them have ~10 % difference in capsid amino acids when compared with the parent rO1K-wt virus. Similarly, both the Cathay topotype viruses and two viruses of different serotypes (A22/IRQ and Asia 1 Shamir) were not neutralized at all when tested against guinea pig antisera, even though the viruses from different serotypes have 20 % more amino acid changes in the capsid region than the Cathay viruses. This implies that the position of the capsid amino acid substitution is more critical than the total number of changes, which is in line with other studies (Ludi *et al.*, 2014; Upadhyaya *et al.*, 2014).

Comparison of the capsid sequence of these viruses in the five previously reported neutralizing antigenic sites (Table 2b) showed that the viruses from a different serotype had amino acid substitutions at most positions, whereas greater conservation was apparent amongst serotype O viruses. However, this ignores the contribution of amino acid changes in the rest of the capsid. The reduction in neutralization of the 5M5 mutant virus was ~80 %, which is double that of the O/ITL/1/93 and comparable to that of the O/UGA/3/2002 and O/PHI/2/95. Further work is necessary to determine the least number of mutations required to make a type O mutant that could completely resist neutralization by polyclonal antibodies.

In conclusion, we have shown that impairment of the five antigenic sites of type O FMDV is not enough to achieve complete escape of virus neutralization using sera raised against wt virus from either guinea pigs or cattle. This study also provides evidence of new neutralizing epitopes that are linked to the previously reported sites in serotype O FMD viruses. Understanding the changes in protective epitopes of the FMD viruses would help immensely in improving our strategies for rational vaccine selection and development. Current methods to select FMD vaccine strains depend upon serological comparisons between viruses, and are laborious and hard to standardize and interpret (Paton *et al.*, 2005). A better understanding of the antigenic role of different features of the FMDV capsid surface could allow more rapid capsid gene-sequencing approaches to be used to predict the importance of evolutionary changes in circulating viruses. This could identify whether the vaccines in use are appropriate, or the need for selecting or developing new strains, as well as informing the design of new more broadly cross-reactive vaccines.

## Methods

### 

#### Viruses and cells.

Three derivatives of the FMD O1 Kaufbeuren (O1K) virus were used in this study. This virus was originally isolated in southern Germany in 1966. The FMDV O1K-wt is a plaque-purified virus that has been passaged three times in IB-RS2 (pig kidney) cells and is the parent virus that was used to derive the four- and five-site mar-mutant viruses described by Crowther *et al.* (1993) and Dunn *et al.* (1998). The four-site mutant virus G67 (Pirbright) was also used in this study. For reverse genetics work, an infectious copy (pT7S3) was used; this contains the B64 strain of O1K (passaged 64 times in BHK-21 cells).

The other serotype O, A and Asia 1 isolates used in this study were obtained from the World Reference Laboratory for FMD (WRLFMD) at Pirbright. Either IB-RS2 or BHK-21 cells were used to grow wt or recombinant viruses. When the CPE was almost complete (generally 18–36 h post-infection), virus stocks were prepared by a single freeze–thaw cycle followed by removal of cell debris by centrifugation at 1280 ***g*** for 10 min. All the viruses were stored at −70 °C until use. IB-RS2 and BHK-21 cells were used for virus titration and electroporation, respectively, to recover virus from cDNA clones.

#### Antigen (146S) preparation.

Sucrose gradient-purified FMDV antigens were prepared for use in ELISA and also for use as vaccine antigen for generation of antisera. The parent and mutant viruses generated in this study were grown on BHK-21 cells. When CPE was complete, the culture supernatant was harvested, clarified by centrifugation and inactivated with 5 mM binary ethylenimine at 25 °C for 24 h (Bahnemann, 1975, 1990). The antigen was concentrated from culture supernatant by polyethylene glycol precipitation before sucrose density gradient purification (Minor, 1986). The purified antigen was stored at −70 °C until use.

#### Polyclonal sera.

Rabbit and guinea pig anti-FMDV polyclonal sera used as trapping or detecting antibodies in ELISA were prepared against purified serotype O FMDV, O1K. These antisera are routinely used in the WRLFMD for the detection of FMDV in an indirect sandwich ELISA, and were raised in rabbits and guinea pigs by immunizing the animals with inactivated, purified 146S FMD virus particles in Freund’s complete and/or incomplete adjuvant as previously described (Ferris *et al.*, 2005). Before use, these antisera were mixed with an equal volume of normal bovine serum to absorb antibodies produced against bovine serum proteins associated with the purified virus inocula, in order to prevent potential cross-reactivity.

In addition, for use in serological assays, antisera were prepared in guinea pigs against the recombinant parent virus recovered from the cDNA clone (rO1K-wt), which is the parent to all other mutant viruses generated in this study. The vaccine was prepared as a water-in-oil-in-water emulsion with Montanide ISA 50 (Sepic) as recommended by the manufacturer (1 : 1 ratio). The guinea pigs were housed in the high-containment isolation facility of the Pirbright Institute and were observed for 7 days before the beginning of the experiment, to ensure they were in good health. At the beginning of the experiment all the guinea pigs were sero-negative for FMDV. A group of nine guinea pigs were injected subcutaneously with 1 ml of an emulsion containing 5 µg of the FMDV antigen. All the animals received a booster dose at 28 days post-vaccination. The experiment was terminated 2 weeks later and all the blood collected for separation of serum. The serum was stored at −20 °C until use. A pool of sera from nine animals was used in serological tests. The O BFS bovine antisera (21 days post-vaccination) used in the micro-neutralization assay were derived from cattle potency tests carried out at Pirbright. A pool of sera from five animals was used for this purpose.

#### Monoclonal antibodies.

The mAbs used in this study had been produced against the O Lausanne or O Manisa vaccine strains (Table 2a). The O Lausanne panel comprised six well-characterized neutralizing mAbs against five antigenic sites (Crowther *et al.*, 1993; Kitson *et al.*, 1990), and the anti-O Manisa mAbs comprised four well-characterized neutralizing mAbs directed against antigenic site 2 (Aktas & Samuel, 2000). Three bovine mAbs raised against O Lausanne virus were also used in this study (Barnett *et al.*, 1998).

#### RNA extraction, RT-PCR and sequencing.

Total RNA from cell culture-grown viruses was extracted using RNeasy Mini kits (Qiagen) as recommended by the manufacturer. Reverse transcription and PCR to amplify the capsid-coding region were carried out essentially as described previously (Mahapatra *et al.*, 2008). Nucleotide sequencing was carried out as described previously (Upadhyaya *et al.*, 2014).

#### Construction of plasmids containing full-length FMDV genomes.

An infectious cDNA (pT7S3) containing the B64 strain of O1K that had been previously described (Zibert *et al.*, 1990) and subsequently modified (Ellard *et al.*, 1999) was used in this study. A unique, naturally occurring *AflII* site at the 3′ end of the L-gene (nt 4327–4332 in the full-length clone, pT7S3) and a *Spe*I site introduced at the beginning of the 5′ end of the 2B-gene (nt position 6798–6804) were used to facilitate substitution of the capsid-coding genes from other viruses (Fig. S2). The pT7S3 cDNA clone originally had a *Spe*I site at nt position 2756–2762 (at the beginning of the vector harbouring the full-length of FMDV); this *Spe*I site was first deleted by inverse PCRs (IPCR) using overlapping primer sets (Table S1) to make the introduced *Spe*I site at the beginning of the 2B-gene unique. In order to introduce the *Spe*I site, the 5 kb *Xba*I digestion product of pT7S3 plasmid (flanking the 5′UTR to the coding region for 3A) was cloned into pGEM9zf (Promega) to make pGEM9zf/FMDV, which was used as a template for introduction of the *Spe*I restriction site by IPCR using overlapping primer sets (Table S1). Final IPCR products were digested with *Dpn*I enzyme and cloned to produce the pGEM9zf/FMDV1 plasmid. The 5 kb *Xba*I digestion product of plasmid pGEM9zf/FMDV1 was used to replace the corresponding fragment of pT7S3 to generate pT7S3-*Spe*I. Infectious virus was recovered from this cDNA clone and was sequenced to confirm the presence of the unique *Spe*I site in the 2B region. This pT7S3-*Spe*I plasmid was subsequently used in all other experiments.

#### Construction of mutant plasmids.

The capsid-coding region within the cDNA plasmid pT7S3-*Spe*I was replaced with that from three other viruses, namely O1K-wt, O/ITL/1/93 and G67 (Fig. S1). O/ITL/1/93 was used as a control to study the threshold of neutralization of virus from a different FMDV O topotype. For this, total RNA was extracted from cell culture-grown viruses of each donor strain, and PCR products of the capsid-coding genes were generated from cDNA templates using appropriate primer sets (Table S1). The PCR products containing the capsid-coding sequences for O1K-wt, O/ITL/1/93 and G67 were cloned into the pT7Blue (Novagen) to produce pT7Blue/O1K-wt, pT7Blue/O/ITL/1/93 and pT7Blue/G67, respectively, using standard cloning procedures. The *AflII* and *Spe*I-digested products of pT7Blue/O1K-wt, pT7Blue/O/ITL/1/93 and pT7Blue/G67 plasmids were inserted into similarly digested pT7S3-*Spe*I to generate pT7S3/O1K-wt, pT7S3/O/ITL/1/93 and pT7S3/G67 plasmids, respectively. In an attempt to block the antibody binding on the surface of FMDV capsid completely, 26 residue positions (Table 1b) were selected to be substituted taking into account (i) previously identified critical residues by mar-mutant and/or related studies, (ii) the surface exposure and (iii) accessibility of the residues to antibody binding. Two versions of the capsid of O1K virus with mutations at 26 residue positions were synthesized commercially (Genescript), one (O1K-wt26n) where the residues were changed to a residue as reported to prevent/influence mAb binding and the second version (O1K-wt26a) where the residues were substituted with alanine (Table 1b). The capsid-coding regions of the pT7S3-*Spe*I plasmid were successfully replaced with the capsid of the commercially synthesized clones to produce pT7S3/O1K-wt26n and pT7S3/O1K-wt26a. For all the plasmids generated in this study, the complete capsid-coding region was sequenced on both the strands to ensure it was from the desired virus and without unintended mutations.

#### Site-directed mutagenesis.

Site-directed mutagenesis was used to introduce mutations at specific positions in the capsid. A five-site mutant (5M) was generated in this study by substituting the critical amino acid residues representing the five neutralizing antigenic sites. G67 had six substitutions (Table 1b) at neutralizing antigenic sites 1–4; therefore a mutation was introduced in plasmid pT7S3/G67 at codon position VP1-149 (cct to act) to make the five-site mutant pT7S3/5M (Table S1). Further mutations in pT7S3/5M either individually or in combination were introduced to make recombinants containing single or double amino acid substitutions in the capsid (Table 1a; Fig. 1a, b). Three residues, VP2-74, VP2-191 and VP3-85, were selected for this purpose as they were indicated to have an impact on the antigenicity of the serotype O virus by comparison of capsid sequences with *in vitro* virus cross-neutralization data and also by epitope prediction using the virus crystal structure. 

#### Electroporation and recovery of infectious recombinant viruses from full-length cDNA clones.

The parent plasmid and its derivatives were linearized by digestion with *Hpa*I enzyme and full-length RNA transcripts were synthesized by *in vitro* transcription with T7 RNA polymerase (MEGAscript; Ambion) as per the manufacturer’s instructions. The RNAs were electroporated into BHK-21 cells using Genepulse (Bio-Rad), essentially as described previously (Seago *et al.*, 2012). Electroporated BHK-21 cells were incubated for 24 h and the appearance of CPE was monitored. Rescued viruses were harvested following one cycle of freezing and thawing, and subsequently passaged at least three times before stocks of viruses were made.

#### Virus characterization.

In order to characterize the recombinant viruses, RT-PCR was carried out on the total RNA isolated from virus-infected BHK-21 cells. The whole capsid was amplified and then sequenced on both the strands to ensure all the mutations were present in the respective viruses. Multistep growth curves and plaque morphology of the parent and recombinant viruses were determined as described previously (Seago *et al.*, 2012).

#### Virus neutralization test.

Tests for FMD virus-specific neutralizing antibodies were carried out in microtitre plates by two-dimensional VN testing using the guinea pig or bovine antisera following established methodology (Rweyemamu *et al.*, 1978). Virus titration plates for each virus (parent and mutant viruses) were also included in each test to control fluctuations in the virus titre. Antibody titres were calculated from regression data as the log_10_ reciprocal antibody dilution required for 50 % neutralization of 100 tissue culture infective units of virus (log_10_SN_50_/100 TCID_50_). The antigenic relationship of viruses based on their neutralization by antibodies is given by the ratio *r*_1_, the neutralizing antibody titre against the heterologous virus/neutralizing antibody titre against the homologous virus. The significance of differences in the values of *r*_1_ obtained by the polyclonal antiserum was evaluated according to the criteria of Rweyemamu & Hingley (1984). All the tests were carried out in duplicate and repeated at least three times; the mean of the tests was used for subsequent analysis.

#### Indirect ELISA for profiling of the recombinant viruses.

An indirect ELISA was carried out to check the binding of anti-FMDV mAbs or guinea pig polyclonal sera to the recombinant viruses, essentially as described previously (Samuel *et al.*, 1991). Briefly, rabbit hyperimmune serum raised to FMDV O1K was used as trapping antibody in carbonate/bicarbonate buffer, pH 9.6 (Sigma), and incubated at 4 °C overnight. Viral antigen was added to the wells as purified 146S antigen or culture supernatants diluted with an equal volume of a buffer consisting of PBS and 0.1 % Tween 20 (Sigma). This was followed by incubation with the mAbs diluted in blocking buffer, 5 % skimmed milk (Marvel; Cadbury) and 0.1 % Tween 20. All tests included a polyclonal guinea pig serum raised to the O1K strain as a positive control to ensure that sufficient virus antigen was present. Bound mAb or polyclonal antibody was detected using a rabbit anti-mouse, anti-guinea pig or anti-bovine horseradish peroxidase conjugate (Dako). The optical density was read at 492 nm and an optical density in excess of three times the background value was considered positive. All the tests were carried out in duplicate and repeated at least twice, and the mean of the tests used for subsequent analysis.

#### Data analysis.

Minitab 16 statistical software (Minitab) was used for the data analysis.
